# Henry Ormerod

**Published:** 1903-06

**Authors:** 


### HENRY ORMEROD.

Henry Ormerod, M.R.C.S. Eng., 1856, L.S.A., 1857, whose decease took place at Westbury-on-Trym, on the 30th of March, at the age of 68, belonged to a family of surgeons. He was educated at the Bristol Royal Infirmary and Medical School, and very soon after qualifying he settled at Westbury. Although his capacity for work was immense, yet in the early years of his practice he had more than one attack of hæmoptysis, and it was his opinion that leading a country open-air life had been the means of warding off more pronounced symptoms of phthisis. Mr. Ormerod was eminently successful as a general practitioner, and, indeed, was one of the best all-round men who ever carried on his profession in our city. He had the capacity, too, for keeping up to the times, and, as far as clinical work was concerned, was an expert microscopist. For many years he would scarcely allow himself a day's holiday, driving about all day, and often at night, in an open vehicle, and in all kinds of weather. He loved his profession, and upon his death-bed he expressed his gratitude that he had lived the life of a doctor, and declared that, had he to choose again, that should be his life's work. Kindness, self-sacrifice, and courtesy were the leading features of his most charming character.

Some eight years ago Mr. Ormerod suffered from functional heart disturbance, accompanied with dyspepsia, which partially disabled him for a time; but he seemed to quite recover from this. For a year or more before his fatal illness—of acute pneumonia—he had gradually lost as much as two stone in weight, and he had a difficulty in walking uphill owing to painful breathlessness. This interfered with his taking any exercise, and also with gardening, which was one of his favourite pastimes. It also to a large extent prevented his pursuit of photography, which he had carried to a high pitch of perfection; indeed, Mr. Ormerod's fame as an amateur photographer was not confined to the neighbourhood in which he lived. His collection was probably one of the finest of its kind. In the Clifton Photographic Society he took the keenest interest, and was a regular attendant at its meetings.

Mr. Ormerod never paid an unnecessary visit, and always discouraged the whims and imaginary ailments of his patients; but in genuine illness no one could be more skilful or attentive, let it be a medical case or a surgical or obstetric operation; and in his well-merited reward he gained the confidence and respect of his medical brethren and patients alike. Mr. Ormerod leaves a widow and three sons, two of whom are in the medical profession and one in the Church.

